# Partial suppression of M1 microglia by Janus kinase 2 inhibitor does not protect against neurodegeneration in animal models of amyotrophic lateral sclerosis

**DOI:** 10.1186/s12974-014-0179-2

**Published:** 2014-10-19

**Authors:** Satoru Tada, Tatsusada Okuno, Yasumichi Hitoshi, Teruhito Yasui, Josephe Archie Honorat, Kazushiro Takata, Toru Koda, Hiroshi Shimagami, Choong Chi-Jing, Akiko Namba, Tomoyuki Sugimoto, Saburo Sakoda, Hideki Mochizuki, Hitoshi Kikutani, Yuji Nakatsuji

**Affiliations:** Department of Neurology, Osaka University Graduate School of Medicine, 2-2 Yamadaoka, Suita, Osaka 565-0871 Japan; Department of Molecular Immunology, Research Institute for Microbial Disease, WPI Immunology Frontier Research Center (IFReC), Osaka University, 3-1 Yamadaoka, Suita, Osaka 565-0871 Japan; Rigel Pharmaceuticals, Inc., 1180 Veterans Blvd., South San Francisco, CA 94080 USA; Department of Mathematical Sciences, Hirosaki University Graduate School of Science and Technology, 3 Bunkyo-cho, Hirosaki, Aomori 036-8561 Japan; Department of Neurology, National Hospital Organization Toneyama National Hospital, 5-1-1 Toneyama, Toyonaka, Osaka 560-0045 Japan

**Keywords:** Amyotrophic lateral sclerosis, SOD1-G93A transgenic mice, R723, Janus kinase 2, JAK2 inhibitor, Neuroinflammation, Interferon gamma, M1/M2 microglia

## Abstract

**Background:**

Accumulating evidence has shown that the inflammatory process participates in the pathogenesis of amyotrophic lateral sclerosis (ALS), suggesting a therapeutic potential of anti-inflammatory agents. Janus kinase 2 (JAK2), one of the key molecules in inflammation, transduces signals downstream of various inflammatory cytokines, and some Janus kinase inhibitors have already been clinically applied to the treatment of inflammatory diseases. However, the efficacy of JAK2 inhibitors in treatment of ALS remains to be demonstrated. In this study, we examined the role of JAK2 in ALS by administering a selective JAK2 inhibitor, R723, to an animal model of ALS (mSOD1^G93A^ mice).

**Findings:**

Orally administered R723 had sufficient access to spinal cord tissue of mSOD1^G93A^ mice and significantly reduced the number of *Ly6c positive* blood monocytes, as well as the expression levels of IFN-γ and nitric oxide synthase 2, inducible (iNOS) in the spinal cord tissue. R723 treatment did not alter the expression levels of Il-1β, Il-6, TNF, and NADPH oxidase 2 (NOX2), and suppressed the expression of Retnla, which is one of the markers of neuroprotective M2 microglia. As a result, R723 did not alter disease progression or survival of mSOD1^G93A^ mice.

**Conclusions:**

JAK2 inhibitor was not effective against ALS symptoms in mSOD1^G93A^ mice, irrespective of suppression in several inflammatory molecules. Simultaneous suppression of *anti-inflammatory microglia* with a failure to inhibit critical other inflammatory molecules might explain this result.

**Electronic supplementary material:**

The online version of this article (doi:10.1186/s12974-014-0179-2) contains supplementary material, which is available to authorized users.

## Findings

### Introduction

Amyotrophic lateral sclerosis (ALS) is a devastating disease characterized by progressive degeneration of motor neurons in the brain and spinal cord, resulting in muscle weakness. Although the precise mechanism of ALS remains unknown, inflammatory microglial activation plays an important role in pathogenesis [1-3]. The inflammatory molecule IFN-γ, which is primarily produced by Th1 lymphocytes and is a potent activating factor for inflammatory M1 microglia, contributes to the loss of motor neurons in ALS [4,5]. Furthermore, a recent report showed that Ly6c-high inflammatory monocytes are recruited to the spinal cord in mSOD1^G93A^ mice, and that treatment with anti-Ly6c monoclonal antibodies reduces monocyte recruitment to the spinal cord and ameliorates neurodegeneration in these animals [6].

Janus kinases (JAKs) are centrally implicated in cytokine receptor-mediated cell signaling pathways, which drive a range of myeloid malignancies [7] as well as inflammatory diseases [8]. The JAK family member JAK2 is responsible for transducing signals for several proinflammatory cytokines including IFN-γ and Il-12, as well as for differentiation of myeloid cells [9]. In an animal model of rheumatoid arthritis (RA) and experimental autoimmune encephalitis (EAE), suppression of the JAK pathway ameliorated disease severity by suppressing Th1 cells and deactivating monocytes [10,11]. A growing number of JAK inhibitors have been developed and clinically applied to the treatment of various inflammatory disease including RA, psoriasis, and inflammatory bowel disease [12-14]. Although activators of JAK2 such as IFN-γ, Il-6, and Il-12 are reported to be implicated in ALS pathogenesis [3], the role of JAK2 in the ALS-related neuroinflammation remains totally unknown.

Based on these findings, we hypothesized that JAK2 inhibition could ameliorate neurodegeneration in ALS model mice by inhibiting harmful inflammatory processes in microglia/macrophages. To test this idea, we treated transgenic mice overexpressing the familial ALS-associated G93A SOD1 mutation (mSOD1^G93A^ mice) with R723, an orally active inhibitor of JAK2 [15].

## Methods

### Ethics statements

All animal experiments were conducted in accordance with the guidelines of Osaka University, which specifically approved this study (Permit number: Biken-AP-H21-28-0).

### RNA extraction and RT-qPCR analysis

Spinal cord tissues were collected from mSOD1^G93A^ mice and total mRNA and cDNA were generated as previously described [1]. The synthesized cDNA was amplified using SYBR Premix Ex Taq II (for TNF, MCP1, Il-12b, iNOS, Il-6, Il-1b, NOX2, Ly6c, Arg1, Ym1, Il-4, EPO, CSF3 and Retnla) (Takara Bio Inc., Otsu, Japan) or TaqMan Gene Expression Assays (for IFN-γ, Il-6, Il-12a and GM-CSF) (Applied Biosystems, Foster City, CA, USA) and analyzed as previously described [1].

### Immunohistochemistry

Spinal cord sections of mSOD1^G93A^ mice were prepared as previously described [1]. The following antibodies were used: rabbit anti-JAK2 (phospho Y1007 + Y1008) monoclonal antibody (1:200; Abcam, Cambridge, UK), rabbit anti-iNOS polyclonal antibody (1:50; BD Biosciences, Franklin Lakes, NJ, USA) and Alexa Fluor 488®-conjugated mouse anti-glial fibrillary acidic protein (GFAP) monoclonal antibody (1:200; Cell Signaling Technology, Beverly, MA, USA). The following secondary antibodies were applied: Cy5-conjugated F(ab’) 2 fragment donkey anti-rabbit IgG (1:500; Jackson ImmunoResearch Laboratories, West Grove, PA, USA).

### Animals and R723 administration

mSOD1^G93A^ mice were obtained from The Jackson Laboratory and backcrossed with C57BL/6 mice for at least 10 generations. R723 was administered by oral gavage starting on day 90. For the analysis of motor function by rotarod test, weight measurement, and survival, R723 dosing continued until day 120 (70 mg/kg twice daily; 5 days on, 2 days off). To evaluate *in vivo* pharmacokinetics, plasma and spinal cord tissues were collected at 0.5, 1, 2, and 4 hours post-dose, and R723 levels in plasma and spinal cord tissue were determined by LC/MS/MS.

### Flow cytometry of peripheral blood cells

Peripheral blood cells were collected from mSOD1^G93A^ mice on day 4 post-dose. The following antibodies were used: APC-Cy7-labeled anti-CD11b (M1/70; BioLegend, San Diego, CA, USA) and fluorescein isothiocyanate (FITC)-labeled anti-Ly6c (HK1.4; BioLegend, San Diego, CA, USA). Flow cytometry was performed using a FACS Canto™ II with the Diva ™ software (Becton Dickinson, Franklin Lakes, NJ, USA). Acquired data were analyzed using the FlowJo software (Tree Star, Inc., Ashland, OR, USA).

### Lectin staining

Sections were permeabilized with 0.2% tris-buffered saline with tween (TBST) for 10 minutes and then incubated with FITC-conjugated tomato (*Lycopersicon esculentum*) lectin (Sigma-Aldrich, St Louis, MO, USA) diluted 1:750 in PBS overnight at 4°C. The sections were washed ×3 in 0.2% TBST for 5 minutes and mounted with VECTASHIELD Mounting Medium containing 4′,6-diamidino-2-phenylindole (DAPI) (Vector Laboratories, Burlingame, CA, USA). The fluorescently labeled sections were examined using a LSM 510 confocal microscope (Carl Zeiss Microscopy, Jena, Germany).

### Nissl staining

Spinal cord sections of mSOD1^G93A^ mice were prepared as previously described [1]. Every fifth section was collected and stained with cresyl violet.

### Statistics

Data are expressed as means ± SEM. Differences in animal weight measurements and rotarod tests were assessed using analysis of variance (ANOVA). Statistical significance in survival experiments was determined using Kaplan-Meier survival statistics. Statistical significance in all other experiments was assessed using the Mann-Whitney *U*-test. *P* < 0.05 was considered statistically significant.

## Results

**Figure 1:**
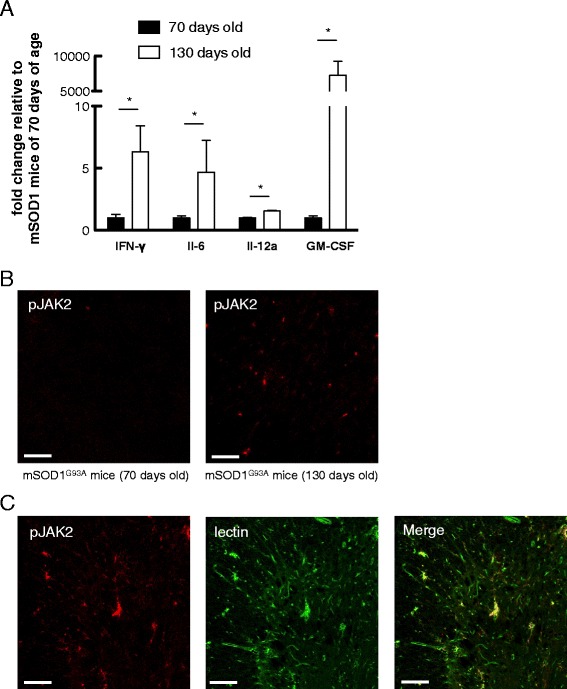
**Enhanced phosphorylation of Janus kinase 2 (JAK2) and up-regulation of JAK2-related genes in the spinal cord of mSOD1** ^**G93A**^ **mice in the late stage of disease. (A)** Quantitative RT-PCR analyses of spinal cords of mSOD1^G93A^ mice (70 days and 130 days old) were performed (n = 3 to 4 for each group). Expression levels of IFN-γ, Il-6, Il-12a, and GM-CSF were significantly elevated in 130-day-old mSOD1^G93A^ mice relative to those in 70-day-old mice. Data are expressed as means ± SEM. *P < 0.05, Mann-Whitney *U*-test. **(B)** Immunohistochemical analysis showed enhanced phosphorylation of JAK2 in the spinal cord of late-stage mSOD1^G93A^ mouse compared with the spinal cord of pre-onset-stage mSDO1 mouse. Scale bar = 100 μm. Data are representative of three animals. **(C)** Sections of 130-day-old mSOD1^G93A^ mouse spinal cord were co-stained with Cy5-conjugated anti-phosphorylated JAK2 antibodies and FITC-conjugated tomato lectin. Scale bar = 100 μm. Data are representative of three animals.

To confirm whether expression of inflammatory cytokines was upregulated in the spinal cords of late-stage mSOD1^G93A^ mice, we evaluated spinal cord mRNA expression of several genes encoding inflammatory molecules. Consistent with a previous report [16], RT-qPCR analysis revealed that the expression levels of IFN-γ, Il-6, Il-12a, and granulocyte macrophage colony-stimulating factor (GM-CSF) increased along with disease progression (Figure 1A and Additional file 1: Supplementary information). In addition, microglia in the spinal cords of late stage mSOD1^G93A^ mice (130 days old) had enhanced phosphorylation of JAK2 compared with pre-onset stage mSOD1^G93A^ mice (70 days old), providing a therapeutic rationale for JAK2 inhibition against ALS (Figure 1B, C).

**Figure 2:**
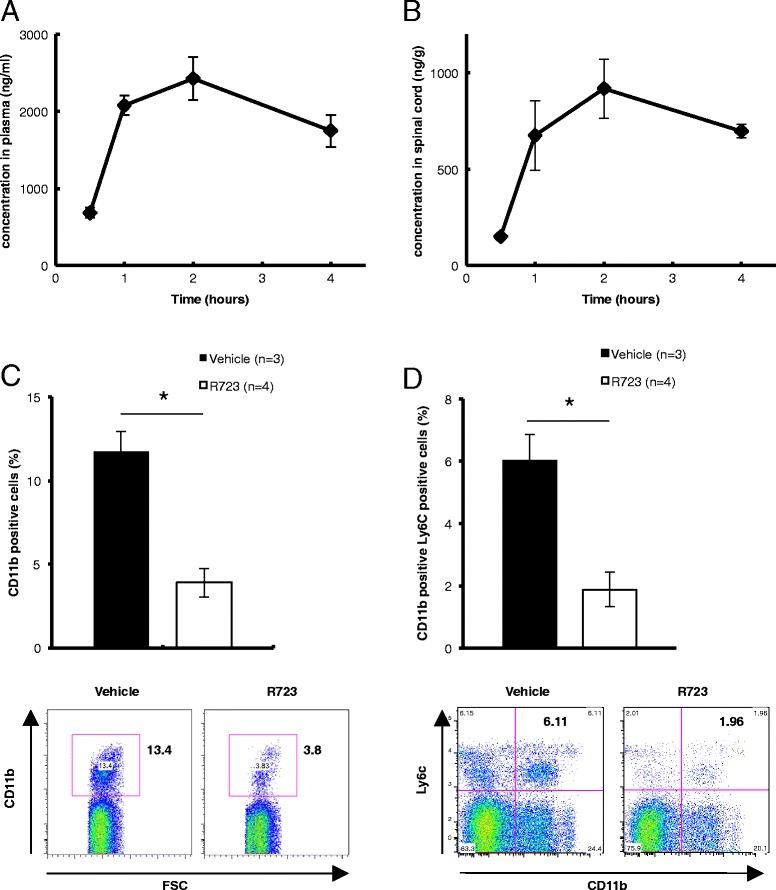
**Pharmacological properties of R723 and its effects on peripheral monocytes. (A, B)** Pharmacological profile of R723 in plasma and spinal cord tissue after single-dose administration by oral gavage to 120-day-old female mSOD1^G93A^ mice. Concentration of the compound at the indicated time points was quantified by LC/MS/MS assay. **(C, D)** Effects of R723 treatment on the proportion of CD11b + or Ly6c + cells in peripheral blood. mSOD1^G93A^ mice (120 days old) were orally administered with vehicle or R723 (70 mg/kg) for 4 days. Blood were harvested, and the proportions of CD11b + and Ly6c + cells were evaluated by FACS. **(C)** The proportion of CD11b + monocytes was significantly reduced in peripheral blood of R723-treated mSOD1^G93A^ mice. **(D)** Four-day treatment with R723 reduced the proportion of Ly6c + CD11b + monocytes in peripheral blood. In panels **(C)** and **(D)**, n = 3 for vehicle-treated mice, and n = 4 for R723 treated mice. Data are expressed as means ± SEM. *P < 0.05, Mann-Whitney *U*-test.

To investigate the role of JAK2 pathway in ALS, we used R723, which is a selective small-molecule JAK2 inhibitor originally developed by Rigel Pharmaceuticals Inc, (San Francisco, CA, USA) for the treatment of myeloproliferative neoplasms such as polycythemia vera, essential thrombocythemia and primary myelofibrosis (Additional file 2: Figure S1A) [15]. First, to investigate the drug distribution, we administered R723 by oral gavage to mSOD1^G93A^ mice and measured concentrations of R723 in serum and spinal cord tissue. R723 had sufficient access to spinal cord tissue (Figure 2A, B) (spinal area under the curve (AUC) (0.5 to 4]/plasma AUC (0.5 to 4] ratio: 0.368) [17]. Next, we tested whether R723 treatment could deplete monocytes circulating in peripheral blood. After 4 days of treatment with R723, mSOD1^G93A^ mice had significantly fewer CD11b-positive cells and Ly6c-positive monocytes in peripheral blood (Figure 2C, D and Additional file 1: Supplementary information).

**Figure 3:**
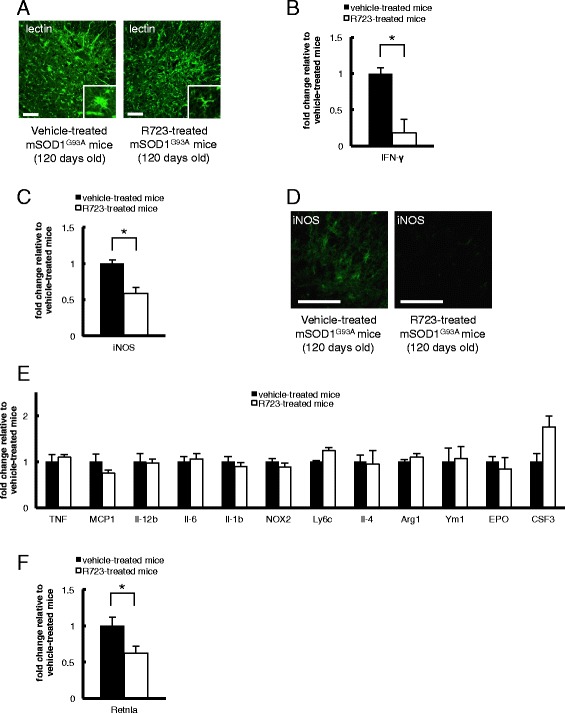
**Effects of R723 on inflammation-related gene expression and microgliosis in spinal cord of mSOD1** ^**G93A**^ **transgenic mice. (A)** R723-treated mSOD1^G93A^ mice had reduced number of lectin-positive microglia in the spinal cord compared with vehicle-treated controls. Lumbar sections of the spinal cord were stained with fluorescein isothiocyanate (FITC)-conjugated tomato lectin. Scale bar = 100 μm. Data are representative of three animals. **(B, C)** Quantitative RT-PCR analyses in spinal cords of R723-treated mSOD1^G93A^ mice and vehicle-treated controls (120 days old) were performed (n = 4 in each group). The expression levels of IFN-γ and iNOS were significantly reduced in the R723-treated group (*P* = 0.0495 for each experiment). **(D)** Immunohistochemical analysis showed R723 treatment for 30 days had suppressed the expression level of iNOS in the spinal cords of mSOD1^G93A^ mice. Scale bar = 100 μm. Data are representative of three animals. **(E)** Quantitative RT-PCR analyses in spinal cords of R723 treated mSOD1^G93A^ mice and vehicle-treated controls (120 days old) were performed (n = 4 in each group). Relative mRNA expression is shown for TNF, MCP1, Il-12b, Il-6, Il-1b, NOX2, and Ly6c, which are related to M1 macrophages/microglia, and for Il-4, Arg1, Ym1, Il-4, EPO and CSF3, which are related to M2 macrophages/microglia. There were no significant differences in the expression levels of these molecules between two groups after the correction of multiple comparisons. **(F)** Quantitative RT-PCR analysis revealed that R723 had suppressed the expression of Retnla after 30 days of treatment in the spinal cords of mSOD1^G93A^ mice (*P* = 0.0495, n = 4 in each group). Data are expressed as means ± SEM. *P < 0.05, Mann-Whitney *U*-test.

To further confirm the anti-inflammatory effect of R723, we evaluated the microgliosis and astrocytosis in spinal cord tissue of R723-treated mSOD1^G93A^ mice. Lectin staining revealed that R723 treatment had suppressed microgliosis in the spinal cords of mSOD1^G93A^ mice, although it did not affect astrocytosis (Figure 3A and Additional file 3: Figure S2A). In addition, we evaluated the mRNA expression of inflammation-related and M1/M2 microglia-related genes in spinal cord tissue of R723-treated mSOD1^G93A^ mice. Consistent with the anti-inflammatory effects of JAK2 inhibitor as previously reported [17], R723 treatment suppressed IFN-γ and iNOS expression dose-dependently, suggesting that the drug exerted anti-inflammatory effects in the spinal cords of mSOD1^G93A^ mice (Figure 3B, C and Additional file 4: Figure S3A). In addition, the effect of R723 against iNOS expression was confirmed by immunohistochemical analysis (Figure 3D). However, R723 had no obvious effects on other inflammatory molecules such as TNF, Il-12b, Il-6, Il-1β, and NOX2. Additionally, there was no significant difference between two groups in the spinal cord expression levels of monocyte chemotactic protein 1 (MCP1) and Ly6c, which are important for the migration and activation of inflammatory monocytes, as well as those of Il-4, arginase, liver (Arg1), chitinase-3-like 3 (Ym1), erythropoietin (EPO), and colony-stimulating factor 3 (CSF3), which are involved in the activation of M2 microglia (Figure 3E). Unexpectedly, R723 suppressed expression of resistin-like alpha (Retnla), a marker of anti-inflammatory M2 microglia, in spinal cord tissue of mSOD1^G93A^ mice after 30 days of treatment, although this effect was not evident after 5 days of treatment (Figure 3F and Additional file 4: Figure S3B). Collectively, these results suggest that oral administration of R723 decreased the number of Ly6c-positive monocytes in peripheral blood and reduced the expression of several inflammatory genes in the spinal cords of mSOD1^G93A^ mice, leading to suppressed microglial activation.

**Figure 4:**
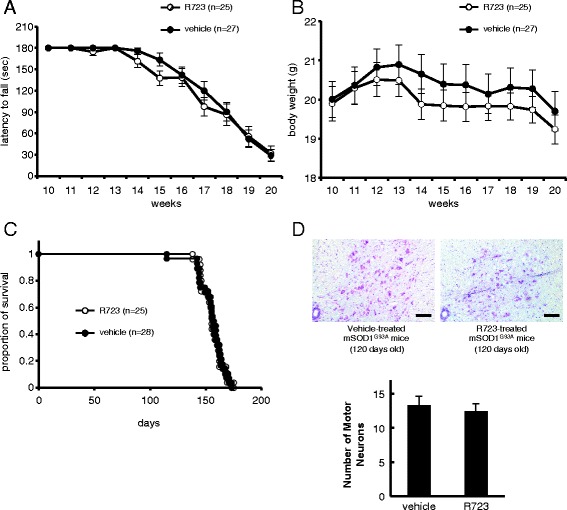
**Body weight, motor performance, and survival of R723- or vehicle-treated mSOD1** ^**G93A**^ **mice. (A)** Time course of motor performance in the rotarod test. Performance of R723-treated (open circles, n = 25) and vehicle-treated (filled circles, n = 27) mSOD1^G93A^ mice were comparable. **(B)** Changes in mean body weight of R723-treated (n = 25) and vehicle-treated (n = 27) mSOD1 ^G93A^ mice. **(C)** Kaplan-Meier survival curve. R723 treatment did not affect the survival of mSOD1 ^G93A^ mice (R723-treated group, n = 25; vehicle-treated group, n = 28) (*P* = 0.964). **(D)** Nissl staining of transverse sections of lumbar spinal cord. Motor neurons were counted and statistical analysis is shown. Scale bar = 50 μm. Data are expressed as means ± SEM. Statistical analysis was performed using ANOVA **(A, B)**, log-rank test **(C)**, and the Mann-Whitney *U*-test.

Because R723 suppresses several pathways that seem to be harmful in ALS, we tested whether R723 could ameliorate neurodegeneration in mSOD1^G93A^ mice. Oral administration of R723 (70 mg/kg, twice daily; 5 days on, 2 days off) to mSOD1^G93A^ mice was started at 90 days of age and continued until 120 days of age. Motor performance was evaluated by rotarod test, and muscle atrophy was monitored by body weight reduction. Decline in motor performance of the R723-treated mSOD1^G93A^ mice was compared with that of the vehicle-treated littermates. Throughout the disease process, there was no significant change in rotarod performance or body weight between the two groups (Figure 4A, B) (*P* > 0.05 for each time point, ANOVA). Additionally, survival times for R723-treated and vehicle-treated mSOD1^G93A^ mice were comparable (Figure 4C) (average survival time; R723 treated group: 155.6 ± 1.8 days (n = 25); vehicle-treated group: 155.1 ± 2.2 days (n = 28), *P* = 0.96, log-rank test). Consistent with these observations, Nissl staining revealed that R723 treatment had led to unaltered motor neuron survival in the spinal cords of mSOD1^G93A^ mice in both groups (Figure 4D). Collectively, these results showed that R723 penetrated the spinal cord of mSOD1^G93A^ mice and suppressed inflammation, but did not affect neurodegeneration *in vivo*.

## Discussion

In this study, we tried to suppress harmful inflammatory processes in mSOD1^G93A^ mice by treating them with a JAK2 inhibitor. Although R723 was effective in depleting Ly6c-positive monocytes and suppressing IFN-γ and iNOS expression in the spinal cord, the drug did not affect disease progression or survival of these mice.

We examined the expression levels of inflammation-related genes including *TNF, MCP1, Il-1β*, and *NOX2*, which play critical roles in the pathogenesis of ALS [3], and found that they were not reduced after JAK2 inhibition. It is possible that inflammation driven by these molecules masked the effects of reductions in IFN-γ and iNOS expression.

One explanation for the lack of a neuroprotective effect of R723 could be the suppression of Retnla, an M2 microglia-related gene. M2 microglial activation, which is driven by Il-4 and Il-13 produced by Th2 lymphocytes, exerts protective roles in ALS [3]. Recently, another group reported that JAK2 is activated after the recruitment of Il-13 to its receptor, and revealed that Il-13 utilizes the JAK2 signaling pathway [18]. Therefore, we speculate that suppression of Retnla counteracts the anti-inflammatory effects of R723, preventing it from exerting a neuroprotective effect *in vivo*.

Alternatively, R723 might have inhibited a neuroprotective effect of JAK2. There is a report that suggests JAK2 signaling is implicated in the prevention of neuronal apoptosis in traumatic brain injury [19].

In conclusion, R723 alone was not sufficient to protect against neurodegeneration in mSOD1^G93A^ mice, although it suppressed the expression of several proinflammatory molecules and depleted monocytes. Based on our results, it is possible that in order to ameliorate neurodegeneration in ALS, we need not only to suppress JAK2 mediated inflammation but also prevent other inflammatory pathways. Furthermore, we may need to activate neuroprotective M2 microglia to alleviate neurodegeneration in ALS.

## Additional files

Additional file 1:
**Supplementary information.** Immunohistochemical analysis of the spinal cord of mSOD1^G93A^ mice and flow cytometric analysis of the peripheral blood monocytes of mSOD1^G93A^ mice. (A) Sections of mSOD1^G93A^ mouse spinal cord were co-stained with FITC-conjugated anti-CD206 receptor antibodies and Cy5-conjugated anti–iNOS antibodies. Scale bar = 200 μm. (B) The number of Ly6c positive and CD11b positive blood monocyte remained unchanged along with the disease progression. Peripheral blood cells were collected from mSOD1^G93A^ mice (70 days old mice and 130 days old ones). The following antibodies were used: APC-Cy7–labeled anti-CD11b and FITC-labeled anti-Ly6c. Flow cytometry was performed using a FACS Canto™ II with the Diva ™ software and acquired data were analyzed using the FlowJo software. Additional file 2: Figure S1. R723 is a selective small-molecule JAK2 inhibitor. (A) Chemical structure of R723 is shown. Additional file 3: Figure S2. R723 had no effect on astrocytosis in the spinal cords of mSOD1^G93A^ mice. (A) The number of GFAP-positive astrocytes in the spinal cord did not differ between R723-treated mSOD1^G93A^ mice and vehicle-treated controls. Lumbar sections of the spinal cord were stained with Alexa Fluor 488®-conjugated anti-GFAP antibody. Scale bar =100 μm. Data are representative of three animals. Additional file 4: Figure S3. R723 had a dose-dependent effect on the suppression of inflammation-related genes. (A) Quantitative RT-PCR analyses revealed that lower dose of R723 (17.5mg/kg, twice a day, 5 days on/2 days off regimen) did not change the expression profiles of IFN-γ, iNOS and Retnla in the spinal cords of mSOD1^G93A^ mice (n = 3 in lower dose group and n = 4 in other groups). (B) Quantitative RT-PCR analyses in spinal cords of R723-treated mSOD1^G93A^ mice and vehicle-treated controls were performed after 5 days of treatment (n = 3 in each group). The expression level of iNOS was significantly reduced in the R723-treated group (*P* = 0.0495). Data are expressed as means ± SEM. **P* < 0.05, Mann-Whitney *U*-test.

## Abbreviations

ALS: amyotrophic lateral sclerosis
ANOVA: analysis of variance
Arg1: arginase, liver
AUC: area under the curve
CSF3: colony-stimulating factor 3
DAPI: 4′,6-diamidino-2-phenylindole
EAE: experimental autoimmune encephalitis
EPO: erythropoietin
FITC: fluorescein isothiocyanate
GFAP: glial fibrillary acidic protein
GM-CSF: granulocyte macrophage-colony stimulating factor
IFN: interferon
Il: interleukin
JAK2: Janus kinase 2
JAKs: Janus kinases
MCP1: monocyte chemotactic protein 1
NOX2: NADPH oxidase 2
PBS: phosphate-buffered saline
PCR: polymerase chain reaction
RA: rheumatoid arthritis
Retnla: resistin-like alpha
TBST: tris-buffered saline with tween
TNF: tumor necrosis factor
Ym1: chitinase-3-like 3
iNOS: nitric oxide synthase 2, inducible
mSOD1^G93A^ mice: transgenic mice overexpressing the familial ALS-associated G93A SOD1 mutation
