# Supplementary material for: Partial suppression of M1 microglia by Janus kinase 2 inhibitor does not protect against neurodegeneration in animal models of amyotrophic lateral sclerosis
Source: J Neuroinflammation. 2014 Oct 19;11:179. doi: 10.1186/s12974-014-0179-2 (PMC4213500; doi:10.1186/s12974-014-0179-2)
Supplement: Additional file 1: — Supplementary information. Immunohistochemical analysis of the spinal cord of mSOD1G93A mice and flow cytometric analysis of the peripheral blood monocytes of mSOD1G93A mice. (A) Sections of mSOD1G93A mouse spinal cord were co-stained with FITC-conjugated anti-CD206 receptor antibodies and Cy5-conjugated anti–iNOS antibodies. Scale bar = 200 μm. (B) The number of Ly6c positive and CD11b positive blood monocyte remained unchanged along with the disease progression. Peripheral blood cells were collected from mSOD1G93A mice (70 days old mice and 130 days old ones). The following antibodies were used: APC-Cy7–labeled anti-CD11b and FITC-labeled anti-Ly6c. Flow cytometry was performed using a FACS Canto™ II with the Diva ™ software and acquired data were analyzed using the FlowJo software. [file 12974_2014_179_MOESM1_ESM.pdf]

Supplementary information

A

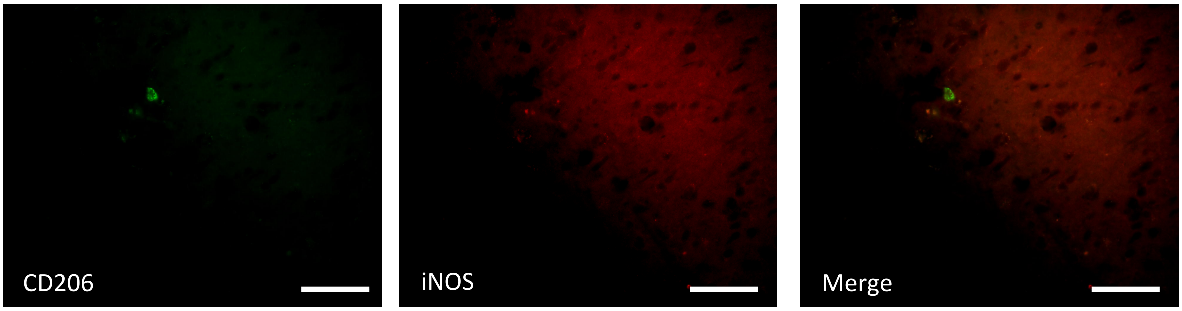

B

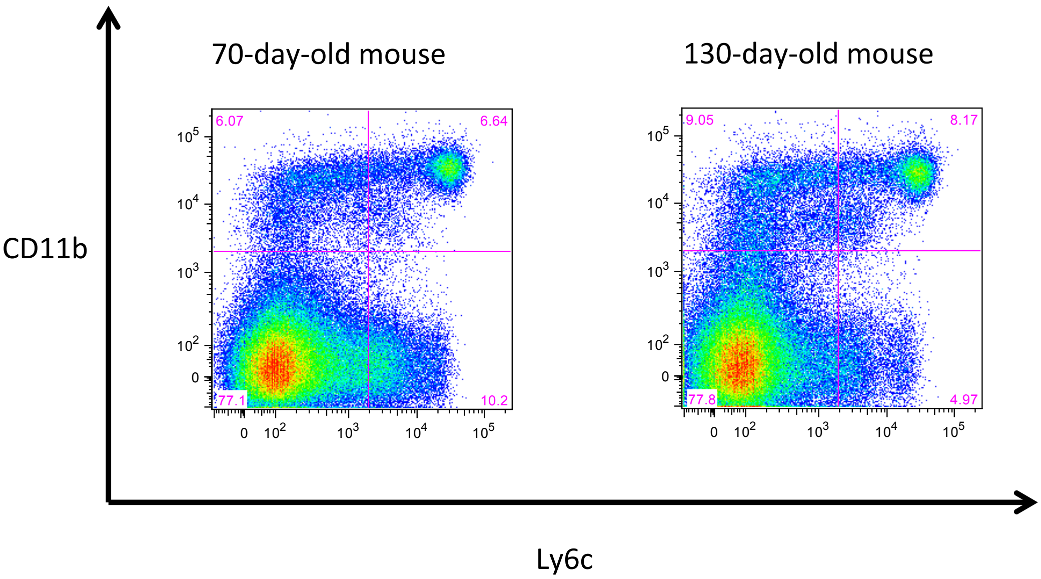

(A) Sections of mSOD1^{G93A} mouse spinal cord were co-stained with FITC-conjugated anti-CD206 receptor antibodies and Cy5-conjugated anti–iNOS antibodies. Scale bar = 200 µm. (B) The number of Ly6c positive and CD11b positive blood monocyte remained unchanged along with the disease progression. Peripheral blood cells were collected from mSOD1^{G93A} mice (70 days old mice and 130 days old ones). The following antibodies were used: APC-Cy7–labeled anti-CD11b and FITC-labeled anti-Ly6c. Flow cytometry was performed using a FACS Canto™ II with the Diva ™ software and acquired data were analyzed using the FlowJo software.
