# Supplementary figures and images for: Partial suppression of M1 microglia by Janus kinase 2 inhibitor does not protect against neurodegeneration in animal models of amyotrophic lateral sclerosis
Source: J Neuroinflammation. 2014 Oct 19;11:179. doi: 10.1186/s12974-014-0179-2 (PMC4213500; doi:10.1186/s12974-014-0179-2)

# Supplementary figure-1

A

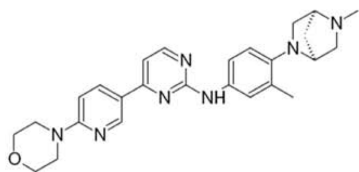

Supplement: Additional file 2: Figure S1. — R723 is a selective small-molecule JAK2 inhibitor. (A) Chemical structure of R723 is shown. [file 12974_2014_179_MOESM2_ESM.pdf]

# Supplementary figure-2

A

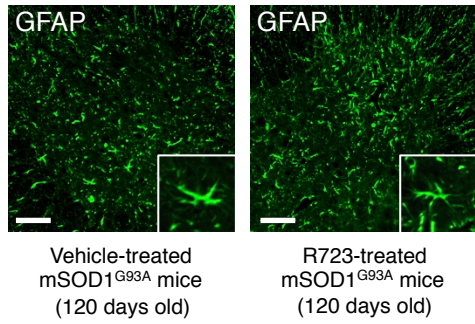

Supplement: Additional file 3: Figure S2. — R723 had no effect on astrocytosis in the spinal cords of mSOD1G93A mice. (A) The number of GFAP-positive astrocytes in the spinal cord did not differ between R723-treated mSOD1G93A mice and vehicle-treated controls. Lumbar sections of the spinal cord were stained with Alexa Fluor 488®-conjugated anti-GFAP antibody. Scale bar =100 μm. Data are representative of three animals. [file 12974_2014_179_MOESM3_ESM.pdf]

# Supplementary figure-3

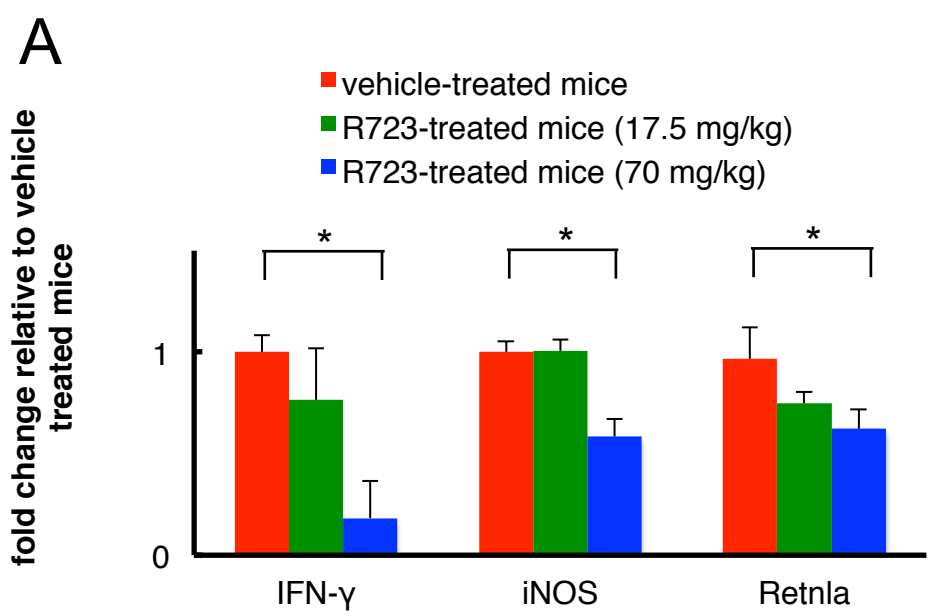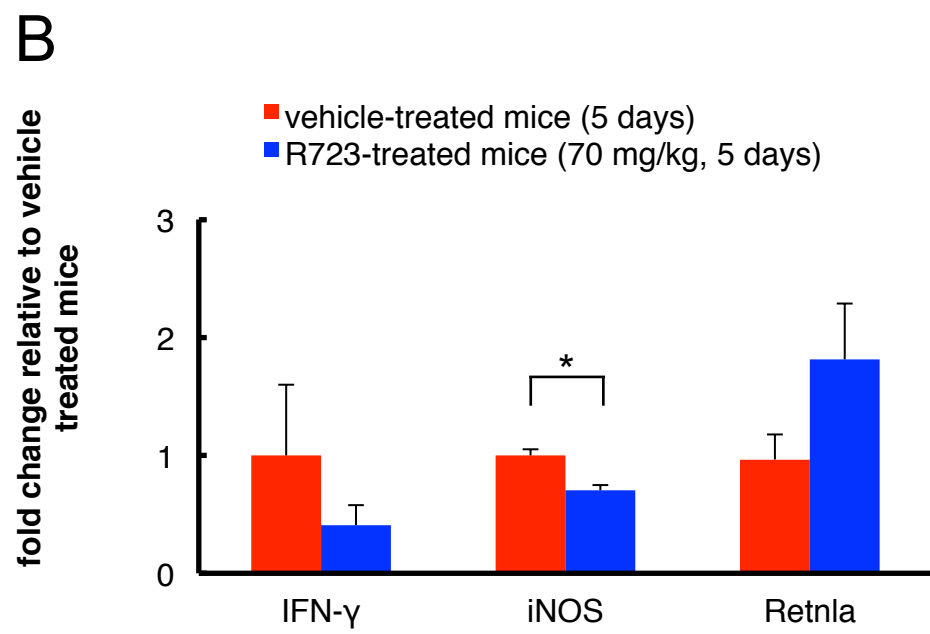

Supplement: Additional file 4: Figure S3. — R723 had a dose-dependent effect on the suppression of inflammation-related genes. (A) Quantitative RT-PCR analyses revealed that lower dose of R723 (17.5mg/kg, twice a day, 5 days on/2 days off regimen) did not change the expression profiles of IFN-γ, iNOS and Retnla in the spinal cords of mSOD1G93A mice (n = 3 in lower dose group and n = 4 in other groups). (B) Quantitative RT-PCR analyses in spinal cords of R723-treated mSOD1G93A mice and vehicle-treated controls were performed after 5 days of treatment (n = 3 in each group). The expression level of iNOS was significantly reduced in the R723-treated group (P = 0.0495). Data are expressed as means ± SEM. *P < 0.05, Mann-Whitney U-test. [file 12974_2014_179_MOESM4_ESM.pdf]
